# Life-threatening abdominal compartment syndrome as a complication of supine super mini percutaneous nephrolithotomy, the first case report and literature review

**DOI:** 10.1016/j.eucr.2021.101578

**Published:** 2021-01-19

**Authors:** Ibrahim A. khalil, Ammar Alani, Ahmed Al Saeedi, Hamzah Shehadeh, Taha Ahmed, Khalid Al-Jalham

**Affiliations:** Department of Urology, Hamad Medical Corporation, Doha, Qatar

**Keywords:** Percutaneous nephrolithotomy, Supermini, Compartment syndrome, Endourology, Life-threatening complication, Supine percutaneous nephrolithotomy

## Abstract

Abdominal compartment syndrome is a life-threatening complication of conventional percutaneous nephrolithotomy (PCNL), with few cases reported in different positions. We present the first case of abdominal compartment syndrome as a complication of supermini percutaneous nephrolithotomy (SMP) in The Galdakao-modified supine Valdivia position, possibly predisposing factors, diagnosis, and management.

Although it is a challenging diagnosis and life-threatening condition, morbidity and mortality can be decreased with early detection and drainage of the intra-peritoneal fluid, causing increased abdominal pressure, which is the most important prognostic factor.

## Introduction

Supermini percutaneous nephrolithotomy (SMP), described by Zeng, uses a mini-endoscopic system with enhanced irrigation and a modified access sheath with a suction-evacuation function that could be used in prone and supine positions. PCNL conventional or SMP are associated with multiple complications, including bleeding, septicemia, or abdominal organ injury. Intra-peritoneal fluid extravasation leading to abdominal hypertension or even abdominal compartment syndrome is a rare complication of PCNL with few case reports. We present the first case of abdominal compartment syndrome as a complication of super-mini percutaneous nephrolithotomy in the modified supine position.

### Case presentation

A 33-year-old gentleman with a history of controlled hypertension and bilateral PCNL admitted for supine PCNL for right lower pole renal stones, measuring approximately 12 x 8 × 13 mm and 7 × 7 × 5 mm. Under general anesthesia, we put the patient in the Galdakao-modified supine Valdivia position. We inserted a ureteric catheter up to a right ureteropelvic junction, followed by puncture of the lower calyx under fluoroscopy by the triangulation method. We had successful access from the first trial, then guidewire inserted down to the ureter followed by co-axial dilator and one step dilatation using SMP sheath. We connected the sheath to the specimen collection bottle with the aspirator, then introduced the nephroscope and started laser dusting and calculi fragmentation and fragments retrieval with irrigation through the nephroscope working channel using a pump.

Fluoroscopy showed residual stone, which was not accessible by the rigid nephroscope. Antegrade flexible ureteroscope introduced through the SMP sheath stone could not be identified. Our total nephroscope time was 2 hours. We fixed a nephrostomy tube through the SMP access.

Decision made for retrograde flexible ureterorenoscopy, but we could not visualize the stone, manipulation of the flexible-ureteroscope was difficult due to abnormal ureter course, so procedure aborted, double-J stent inserted, at the end of the procedure we noticed tense distended with a discrepancy between input and output of irrigation fluid recorded. Bedside ultrasound showed excessive fluid in the peritoneal cavity. The anesthesia team reported difficulty in ventilation and increased airway pressure to more than 20 mmHg. VBG showed metabolic acidosis with high lactic acid.

Immediate drainage of the intra-peritonea through a small incision at McBurney point 3 L of clear fluid drained directly, 16 French Nelaton Catheter fixed for continuous drainage, the patient shifted incubated to SICU on inotropic support.

SICU team experienced ventilation difficulty and high airway pressures (23–25 mmHg), with high intra-abdominal pressure (20 mmHg). Ultrasound revealed residual peritoneal fluid. Patient shifted back to theatre for drain change, right lower abdomen wound re-opened, Nelaton catheter removed, around 400 ccs of clear fluid drained then a 15 fr Jvac drain inserted and fixed, intraoperatively airway pressure improved immediate, and workup showed improvement of acidosis see Table 1.

**Table 1:** Postoperative laboratory results.

Test	Reference range	Immediate post second operation	1st postoperative day	2nd postoperative day	3rd postoperative day	4th postoperative day
WBC (cell/ul)	4–10 x 10^3	17.8 x10^3	12.9 x 10 ^3	12.1 x 10^3	13.2 x 10^3	11.5 x 10^3
Hgb (mg/dL)	13–17	12	10.8	11.2	12.5	12.5
Cr (umol/l)	62–106	119	109	102	96	82
PH	7.35–7.45	7.15	7.33	7.4	7.4	–
HCO_3_ (mmol/L)	22.5–26.9	13.4	19.6	22.9	24.6	–
Lactic acid (mmol\L)	0.36–1.6	6.1	1.2	0.6	0.6	–

The patient had a smooth postoperative course, extubated on the first postoperative day. Follow-up abdominal ultrasound showed no intra-peritoneal free fluid. We removed the drain and discharged the patient.

## Discussion

The abdominal compartment syndrome (ACS) represents the pathophysiologic consequence of abdominal hypertension, which is defined as intra-abdominal pressure (IAP) of 12 mmHg or more. ACS develops when IAP elevates over 20 mmHg and results in abdominal distention, respiratory insufficiency, increased central venous pressure, and decreased urine out. Various clinical conditions associated with this syndrome include massive intra-abdominal or retroperitoneal hemorrhage, severe gut edema or intestinal obstruction, and ascites. Intra-abdominal hypertension precedes ACS. Therefore, ACS is preventable given a timely and appropriate intervention that decreases morbidity and mortality.^1^

In our case, we used SMP in The Galdakao-modified supine Valdivia position, complicated with Intra-abdominal irrigation fluid extravasation that led to ACS, which is an extremely rare complication of PCNL.

Multiple factors resulting in extravasation of irrigation fluid to the intraperitoneal cavity resulting in ACS have been proposed. Ozer et al. reported difficulty during the dilation through the abdominal wall and renal parenchyma.^2^ In contrast, Tao et al. attributed the fluid extravasation to a considerable amount of irrigation fluid used to clear the operative field, operative time of more than 1 hour, and renal pelvis mucosal tear.^3^ Peterson et al. and Ghai et al. proposed that the renal pelvis's accidental perforation caused the fluid extravasation.^4^^,^^5^

In our case, ACS resulted from intra-abdominal extravasation of the irrigation fluid used during SMP. The factors that resulted in the intra-abdominal extravasation were not clear as no tear in the collecting system was identified during the procedure; nevertheless, we encountered several predisposing factors that contributed to fluid extravasation, which are the previous history of PCNL, which might lead to scarring and fibrosis might cause peritoneal tear during dilatation. Long nephroscope time more than 90 minutes, the irrigation-suction sheath position at the lower calyx entry (Just outside the collecting system), and the pump device used in SMP, which increased the amount and pressure of irrigation fluid.

Early detection of intra-abdominal extravasation and development of intra-abdominal hypertension can prevent morbidity and mortality. Increased Airway peak pressure and hemodynamic changes, along with discrepancy between records of irrigation fluid used and output through the suction sheath, should raise the suspicion of increased IAP. Abdominal distention might be evident in patients in the supine position, as in our case. However, it is difficult to detect in the prone position until the patient in the supine position again. We used bedside abdominal ultrasound readily available in our theater, which confirmed that intra-abdominal fluid presence resulted in ACS. Prompt drainage of the abdominal cavity will decrease IAP and airway peak pressure, improving blood return, and hemodynamic stability. Multiple drainage methods were used in the reported cases see Table 2. In all cases, the most important prognostic factor was early detection and proper drainage.

**Table 2:** Literature review of case reports of abdominal compartment syndrome post PCNL.

Author	Patient age	Surgical position	Method of diagnosis	Predisposed factor	Management	Prognosis
Tao et al.	77	Prone	Abdominal ultrasound	Renal pelvis mucosal tear Usage of a large amount of irrigation fluid	Drain insertion in right upper quadrant under ultrasound guidance	Total recovery Extubated day 1 post-operation
Tao et al.	63	Prone	Abdominal ultrasound	Renal pelvis mucosal tear Usage of a large amount of irrigation fluid	Drain insertion in right upper quadrant under ultrasound guidance	Total recovery Extubated day 1 post-operation
Ozer et al.	30	Prone	Inspection and palpation	A technical error in the dilation	Peritoneal lavage	Total recovery Extubated directly post-operation
Etemadian et al.	46	Prone	Diagnostic peritoneal tap	Ruptured thin renal cortex	Abdominal drain insertion followed by exploratory laparotomy	Total recovery
Sharma et al.	29	Prone	Abdominal Ultrasound	–	Drain insertion under ultrasound guidance	Total recovery Extubated directly post-operation
Peterson et al.	25	Lateral Semi prone	Inspection and palpation	perforation of the renal pelvis	Exploratory laparotomy	massive gastrointestinal and retroperitoneal bleeding patient died
Ghai et al.	43	Prone oblique	Abdominal paracentesis	perforation of the renal pelvis	Drain insertion in left lower quadrant under local anesthesia	Totally recovered Extubated directly post-operation

## Conclusion

Although rare, PCNL can be complicated by life-threatening abdominal compartment syndrome due to irrigation fluid extravasation to the peritoneal cavity. Multiple causes have been implicated, from which the renal pelvis tear is the most common. This challenging diagnosis needs a high index of suspension. Abdominal ultrasound has good diagnostic value. The key prognostic factor is early detection and drainage of fluid.

## Declaration of competing interest

The authors declare that they have no financial or non-financial conflicts of interest related to the subject matter or materials discussed in the manuscript.

## Abbreviations

SMP: Supermini percutaneous nephrolithotomy
PCNL: Percutaneous nephrolithotomy
ACS: Abdominal compartment syndrome
IAP: Intraperitoneal pressure
FiO_2_: Fraction of inspired oxygen
VBG: Venous blood gases
ABG: Arterial blood gases
SICU: Surgical intensive care unit
